# A Review on Notch Signaling and Colorectal Cancer

**DOI:** 10.3390/cells9061549

**Published:** 2020-06-25

**Authors:** Ashish Tyagi, Arun K. Sharma, Chendil Damodaran

**Affiliations:** 1 Department of Urology, University of Louisville, Louisville, KY 40202, USA; ashish.tyagi@louisville.edu; 2 Department of Pharmacology, Penn State Cancer Institute, Penn State College of Medicine, Hershey, PA 17033, USA; asharma1@pennstatehealth.psu.edu

**Keywords:** notch signaling, colorectal cancer, negative regulatory region

## Abstract

Colorectal cancer (CRC) has one of the highest mortality rates despite the advancement of treatment options. Aggressive CRC remains difficult to treat owing to the activation of oncogenic signaling pathways such as the Notch signaling pathway. The role of Notch receptors varies according to the difference in their structures; in particular, aberrant activation of Notch1 has been attributed to the severity of CRC. Notch1 activation in CRC is inhibited by small molecule inhibitors that target γ-secretase, an enzyme responsible for the third and last cleavage step of Notch receptors. γ-Secretase also produces the intracellular domain that finally carries out cellular functions by activating downstream effectors. However, most inhibitors block γ-secretase non-selectively and cause severe toxicity. Plant-source-derived small molecules, monoclonal antibodies, biological molecules (such as SiRNAs), and compounds targeting the Notch1 receptor itself or the downstream molecules such as *HES1* are some of the options that are in advanced stages of clinical trials. The Negative Regulatory Region (NRR), which plays a central role in the transduction of Notch1 signaling in the event of ligand-dependent and ligand-independent Notch1 processing is also being targeted specifically by monoclonal antibodies (mAbs) to prevent aberrant Notch1 activation. In this review, we discuss the role of Notch1 in CRC, particularly its metastatic phenotype, and how mutations in Notch1, specifically in its NRR region, contribute to the aberrant activation of Notch1 signaling, which, in turn, contributes to CRC pathogenesis. We also discuss prevailing and emerging therapies that target the Notch1 receptor and the NRR region, and we highlight the potential of these therapies in abrogating Notch signaling and, thus, CRC development and progression.

## 1. Introduction

Colorectal cancer (CRC) is the second most commonly diagnosed cancer in women and the third most common in men. Annually, approximately 53,200 Americans die of CRC, accounting for approximately 8% of all cancer deaths [1]. Despite an apparent decrease in CRC-related morbidity in the past decade, metastatic CRC remains difficult to treat and thus has high mortality rates [2,3]. Therefore, it is imperative to develop treatment strategies that effectively target metastasis and/or its preliminary or initiating stage, i.e., epithelial to mesenchymal transition (EMT). A critical preliminary step in EMT is the development of cellular attributes that lead to changes in the migratory and invasive characteristics of tumor cells, which dissociate and migrate from their originating site and metastasize in distant parts of the human body [4].

Recent studies suggest that compared to other Notch receptors, Notch1 activation is responsible for the aggressive induction of phenotypic and functional changes in cancer cells consistent with mesenchymal transformation. These changes are brought about by direct regulation of Slug and Snail, which finally leads to changes in E-Cadherin and other mesenchymal markers [5], ultimately leading to EMT. Moreover, Jagged-1 ligand-mediated activation of Notch1 in epithelial cells is known to induce a similar mesenchymal transformation, suggesting that Jagged-1-mediated activation of Notch1 signaling is important during the induction of EMT [5].

The structure and function of Notch1 in the normal gastrointestinal system have been well documented [6,7,8]. Notch1 and its target gene, hairy-enhancer-of-split (*HES1*), are expressed more in advanced colon tumors than in low-grade tumors [9]. Recently, we and others have shown that NOTCH1 signaling is key to CRC progression and should be exploited clinically, especially in patients already diagnosed with high-grade polyps or localized colon cancer [10,11]. Hence, this review focuses on the role of Notch1 in CRC and therapies that target different Notch1 signaling by inhibiting Notch1 activation in CRC.

## 2. Notch Receptors: Structure and Function

It is important to understand the structure of Notch proteins and the related signaling pathways that are involved in the regulation of the promotion, proliferation, and progression of cancer. The Notch receptors Notch1 to 4 are transmembrane glycoproteins. Notch receptors are composed of an extracellular domain and a transmembrane domain, followed by an intramembrane or cytoplasmic region. The extracellular domain consists of 33 to 36 tandem epidermal growth factor (EGF) repeats followed by the Negative Regulatory Region (NRR), which includes cysteine-rich LIN-12/Notch-related region (LNR) and two heterodimerization domains (HD-N and HD-C). The NRR plays a critical role in preventing receptor activation in the absence of ligand. Most surface Notch proteins are cleaved by furin-like convertases at site 1 (S1) located within an unstructured loop protruding from the heterodimerization (HD) subdomain, thereby converting the Notch polypeptide into a Notch extracellular domain-Notch transmembrane and intracellular domain heterodimer held together by non-covalent interactions between the N- and C-terminal halves of HD. S1 cleavage likely occurs in the secretory pathway as secreted NRR modules undergo S1 cleavage [12].

**Figure 1:**
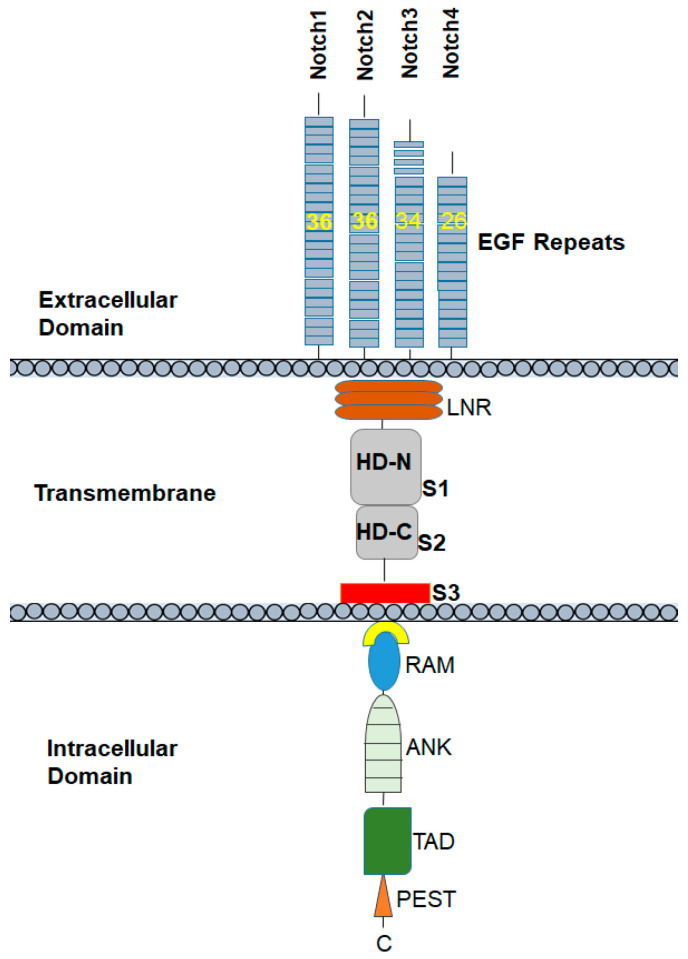
Notch1 receptor structure: the Notch1 receptor has 36 epidermal growth factor (EGF)-like repeats followed by three cleavage sites S1–3, and mutation hotspot regions in the heterodimerization (HD) and proline, glutamine, serine, and threonine residues (PEST) domains.

The intramembrane or cytoplasmic region of Notch contains a Recombination Signal-Binding Protein 1 for the J-kappa (RBP-J)-association molecule (RAM) domain; ankyrin (ANK) repeats, nuclear localization signals (NLS); a transactivation domain (TAD); and a region rich in proline, glutamine, serine, and threonine residues (PEST) sequence (Figure 1). The two most important Notch ligands, Jagged-1 and Jagged-2, are Serrate-like ligands, named after their similar analogues, Delta and Serrate in Drosophila melanogaster. The activation of Notch is initiated when these ligands bind to an adjacent Notch receptor between two neighboring cells. Upon activation, Notch is cleaved, releasing the Notch intracellular domain (NICD) through a cascade of proteolytic cleavages by the metalloprotease, tumor necrosis factor-α-converting enzyme (TACE) and γ-secretase. In the first, operated by the metalloproteinase ADAM 17/TACE, the extracellular domain of the Notch receptor is removed; the second, carried out by the γ-secretase complex (containing presenilin-1/2, nicastrin, PEN-2, and Aph-1), cleaves the NICD, which then translocate into the nucleus. The NICD subsequently binds to transcriptional factors such as *HES1* and mastermind-like-1 (MAML-1), which lead to the activation of downstream pathways.

Notch receptors have been shown to be involved in several developmental processes, such as neurogenesis, somitogenesis, and angiogenesis [13,14]. Transforming growth factor beta (*TGFβ*)-mediated EMT also requires active Notch in the developing heart [15]. Additionally, it is essential signaling for the generation of T cells and its regulation determines the normal or carcinogenic fate of immune cells [16]. Notch receptors have also been shown to regulate the differentiation of colonic goblet cells and stem cells and to redirect the gut progenitor cells to differentiate, not into the lineage cells with secretory attributes, but toward gaining an absorptive attribute [17]. Thus, Notch receptors play a vital role in regulating the proliferation of crypt progenitor cells and the differentiation of colonic epithelial cells. This fine adjustment of cellular attributes maintains intestinal homeostasis and is critical to its development [18,19,20]. It is important to note here that different Notch receptors have demonstrated affinity toward specific cancer types and also function distinctly based on subtle variations in their structure or otherwise on the strength of their activation signals.

## 3. Notch Receptors Have Structural and Functional Differences

The Notch family of receptors includes four types of receptors, classified as Notch1, Notch2, Notch3, and Notch4 receptors. Notch1 and Notch2 are expressed widely in many tissues throughout development and in adult mammals. By contrast, Notch3 is most abundant in vascular smooth muscle and pericytes [21], and Notch4 in the endothelium [22]. These Notch receptor variants have subtle differences in their extracellular and cytoplasmic end regions. The extracellular domains of Notch proteins have multiple epidermal growth factor (EGF)-conserved repeats that are the actual site of ligand binding. Specifically, the Notch1 and Notch2 proteins contain 36 arranged repeats of the EGF-like domain, while Notch3 and Notch4 have 34 and 29, respectively. The cytoplasmic region of Notch receptors—specifically, Notch1 and Notch2—has only a 53% amino acid similarity [23]. As in the extracellular and cytoplasmic domains, NRR also differs in Notch1 and Notch2. The HD-N, HD-C, and LNR-C junction contains a hydrogen bond in the Notch1 NRR, whereas a Zn2+ coordination site is present in the Notch2 NRR. In Notch1, there is an intrahelical salt bridge and the helix is anchored to LNR-C via a single salt bridge. In Notch2, there are several electrostatic interactions between the helix and LNR-C. The LNR A-B linker of the Notch1 and Notch2 NRRs, a three-residue sequence from the protective plug (to protect S2 cleavage), is not conserved [24].

These structural differences make the functions of the Notch receptor variants rather distinct. For example, the Notch1 intracellular domain (N1ICD) is a potent activator of the Hes1 promoter, while the Notch3 intracellular domain (N3ICD) is a much weaker activator and can repress N1ICD-mediated HES activation in certain contexts. Studies have suggested that the differences in outcome segregating Notch1 or Notch2 are likely to reflect outcomes dependent on the overall strength of Notch activation [23]. Also, Notch1 and Notch2 have been correlated with opposite clinical outcomes in colorectal cancer (CRC). Notch1 has been demonstrated to positively predict poor overall survival, while Notch2 negatively predicts poor overall survival [25]. Thus, Notch1 has been shown to promote invasiveness by activating several pro-oncogenic factors, including *CD44*, Cyclin D1 (*CCND1*), and BCL2 Apoptosis Regulator (*BCL2*). The distinct pro-oncogenic and metastatic character of Notch1 makes it a valuable target for designing therapeutic interventions. Among the Notch receptor family (Notch1–4), Notch1 plays a major role in colon cancer. Hence, in this review, we focus only on Notch1 function in CRC.

## 4. The Role of Notch1 in the Proliferation and EMT

A series of recent studies has revealed that both proliferation and apoptotic events can be affected by Notch1 signaling [26]. Activated Notch1 repressed p27 to promote cell cycle and proliferation in cancers such as prostate cancer, adenoid cystic carcinoma, and breast cancer [27,28,29]. Constitutively activated forms of Notch1 have been shown to inhibit Nuclear receptor 77 (*NUR-77*)-dependent apoptosis in T cells [30]. Meanwhile, the knockdown of N1ICD resulted in the inhibition of the proliferation of different cancer cell types, including, but not limited to, ovarian cancer, thyroid cell cancer, and tongue cancer [31]. The inability to undergo apoptosis through physiological mechanisms and resistance to therapeutically induced apoptosis are well-recognized features of the transformed phenotype in many human malignancies [32]. Aberrant Notch signaling was also observed in small-cell lung cancer [33], neuroblastoma [34], cervical carcinoma [35], ovarian carcinoma [36], and prostate carcinoma [37]. Several lines of evidence now suggest that aberrant Notch1 activation or expression contributes to tumorigenesis [38]. However, the distinct functions of different receptors, as mentioned in the section above, have resulted in the suppression or promotion of tumors depending on the cell type and context [39,40,41]. The first evidence of Notch’s involvement in tumorigenesis was noticed when a small subset of T cell acute lymphoblastic leukemia (T-ALL) was found to have constitutive activation of Notch signaling due to a chromosomal translocation of the mammalian NOTCH1 gene [42]. Due to continuous research efforts on Notch receptors, it is now well known that Notch1 mutations are sources of over half of all T-ALL cases [43].

Increased stemness in tumors has been attributed to EMT and several cancer models have identified EMT as the initiating trigger of metastasis [41,44,45,46]. Even the Notch1 ligand Jagged-1 has been frequently reported to be involved in metastasis in prostate, breast, and colon cancers and the activation of NOTCH1 signaling has been shown to directly contribute to cancer cell stemness and invasion in these cancers [47,48]. Elfriede et al. [49] successfully showed that sustained Notch1 activity in epithelial cells is responsible for the development of a senescence-like phenotype, which ultimately enables transmigration of tumor cells within the primary tumor and homing at distant sites. Similarly, the over-expression of Notch1 in immortalized endothelial cells induces Snail expression, decreases E-cadherin expression that finally culminates in the loss of contact inhibition and the acquisition of EMT followed by oncogenic transformation [15]. EMT is induced by Jagged1 by activating Notch1, which, in turn, regulates Slug, which is responsible for EMT. The inhibition of Notch1 has been shown to reverse the Jagged1-induced EMT process in human breast cancer cells [46]. Jagged1-induced Notch signaling activation resulted in the inhibition of E-cadherin expression that impairs cell–cell adhesion and a simultaneous increase in N-cadherin, vimentin, and nuclear localization of β-catenin, which ultimately resulted in an invasive and mesenchymal phenotype [50]. In breast cancer, the rate of N1ICD-positive versus negative tumor epithelial cells (80% ± 10.3% versus 50% ± 12.5%) was correlated with patients who had positive sentinel lymph nodes. In melanoma, N1ICD expression was significantly correlated with higher rates of metastasis (stage IV tumors) and shorter progression-free survival compared to patients with low N1ICD expression [49]. These studies suggested that Notch1 activation plays a major role in EMT and, when activated in the vasculature, presents a major risk factor for metastasis.

## 5. Notch1 Signaling in Colorectal Cancer

As discussed above, Notch signaling has been shown to play a critical role in the maintenance of the normal intestinal epithelia [20]. At the same time, aberrant activation of Notch1 has been shown to initiate CRC. Fre et al. [51] showed that normal Wnt signaling was necessary for the proliferative effect of Notch signaling on early intestinal precursors. In fact, Notch and Wnt signals work together to induce intestinal adenomas, particularly in the colon. The study underlined the potential role of Notch1 activation as an essential initial event triggering colorectal cancer [51]. In some cases, particularly inflammation-induced carcinogenesis, Matrix metallopeptidase 9 (*MMP9*) upregulation in CRC cells has been linked to Notch1 activation [52]. Other studies have shown the absolute necessity of Notch activation for promoting vasculogenesis in intestinal tumors [53]. Over 22% of CRCs report a high copy number gain of the Notch1 receptor that causes tumor cell-autonomous signaling with negative prognostic value [54]. Gene array analysis has shown that Notch1 and its target *HES1* were significantly higher in advanced tumors than in low-grade tumors [9]. Another study confirmed active Notch activation in colon tumors via in situ hybridization [55]. We have shown that the overexpression of *AKT* and thus Notch1 signaling increase CRC cell proliferation and tumor burden [11].

CRC aggressiveness is associated with Notch1-induced EMT. The active role of Notch1 in EMT is due to the close interaction of Notch1 with transcription factors such as *SLUG*, *SNAIL*, and *TGF-β*, which governs EMT. This close association with transcription factors creates a tumor microenvironment that facilitates metastasis in CRC [56]. Similarly, the constitutive activation of N1ICD in CRC cells resulted in increased expression of the EMT/stemness associated proteins CD44, Slug, and Smad-3, and resulted in phenotypic changes in CRC [57]. In addition, crosstalk between Notch receptors and associated ligands has been attributed to EMT in CRC. Notch1 regulates Jagged-1 function, which, in turn, activates Notch3, resulting in increased expression of *SLUG* and CD44 [57]. This leads to EMT and stem-cell-like phenotypes in CRC. Of newly diagnosed CRC patients, 40–50% will develop metastasis; based on the evidence that Notch1 promotes tumorigenesis and the spread of metastatic disease in CRC, targeting Notch1 signaling gains momentum for the treatment of CRC.

## 6. Small Molecule Inhibitors of Notch1 Signaling

Proteolytic processing plays a vital role in the transduction of Notch signals from the extracellular to the intracellular side of the cell. As we have already discussed, this proteolytic processing takes place in three steps. First, a furin-like convertase matures the protein. Second, the binding of ligands activates the Notch receptor that capitulates into a second cleavage (S2 cleavage) by a membrane-tethered metalloprotease (ADAM) which cleaves the ectodomain a second time close to the membrane. The remaining membrane-bound fragment becomes, by default, a γ-secretase substrate. As γ-secretase is the enzyme that is responsible for the release of NICD after it is marked for proteasomal degradation by the E3 ubiquitin ligases Numb and Itch, most of the Notch signaling inhibition research has been focused on gamma secretase inhibitors (GSIs).

**Figure 2:**
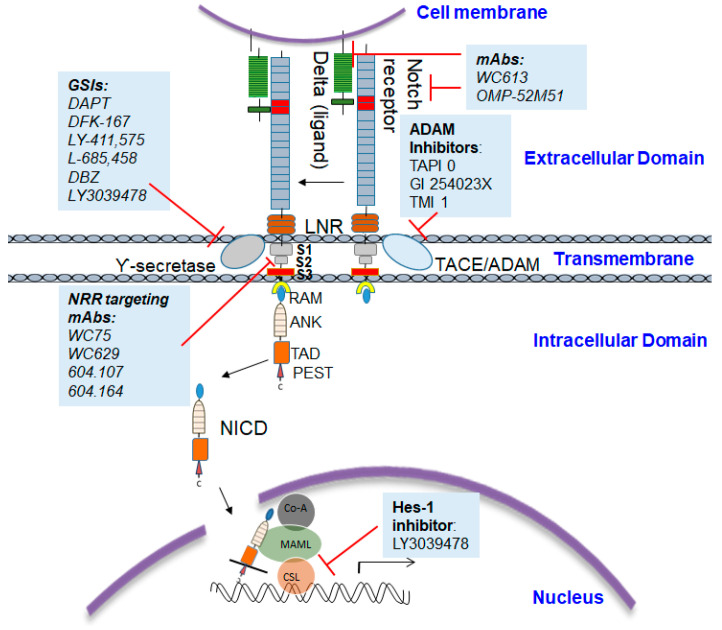
Sites of gamma secretase inhibitors’ (GSIs) binding in γ-secretase: Transition-state analogs such as difluoroketone-167 (DFK-167) bind to catalytic site and small molecule inhibitors such as *N*-[*N*-(3,5-difluorophenacetyl)-l-alanyl]-*S*-phenylglycine t-butyl ester (DAPT) that bind to sites other than catalytic site.

Depending on the structure and binding sites, GSIs can be classified into two types: (1) aspartyl proteinase transition-state analogs as peptide isosteres that mimic the transition state of a substrate cleavage by γ-secretase and bind competitively to the catalytic active site of presenilins; and (2) small molecule inhibitors in which the binding site is different from the active site, possibly at the interface of the γ-secretase complex dimer. The first kind of inhibitors interacts well with the two aspartates in the active site but is not susceptible to cleavage by the protease (for example, difluoro ketone peptidomimetic inhibitors such as difluoroketone-167 (DFK-167) [58]) and binds directly to the active site, while the second type of inhibitors, such as N-[N-(3,5-difluorophenacetyl)-L-alanyl]-S-phenylglycine t-butyl ester (DAPT), LY-411,575, and the clinical candidate LY-450,139, binds to sites different from the active site or docking sites and consists of non-competitive inhibitors of γ-secretase (Figure 2). These inhibitors block the S3 cleavage of Notch receptors to inhibit Notch signaling activation [59,60].

**Figure 3:**
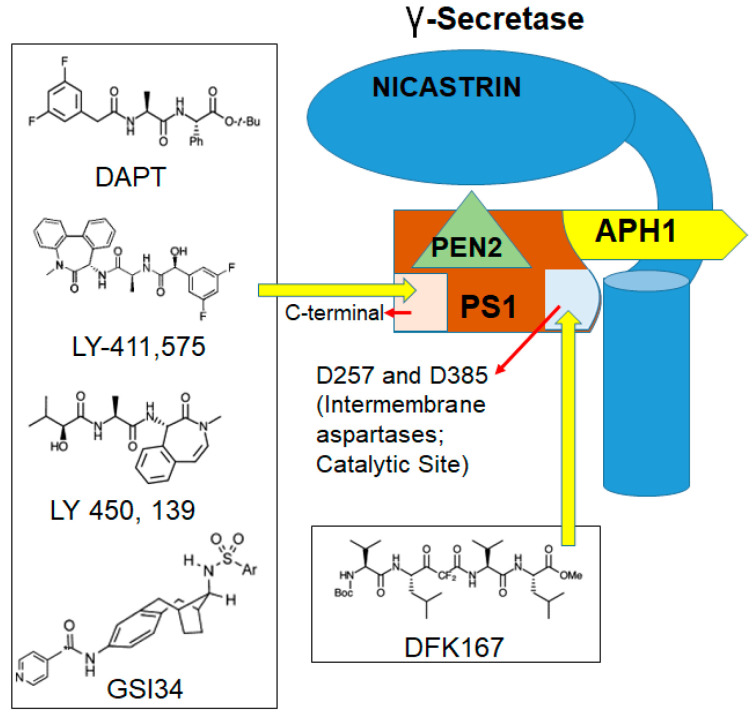
Model for the major events in the Notch signaling pathway: Signals initiated by the engagement of the ligand lead to metalloprotease (MP) cleavage at site S2. γ-Secretase complex cleaves Notch1 at the S3 site within the transmembrane domain. Intracellular Notch (NICD) is released from the membrane, translocates to the nucleus, and forms a complex with CSL and MAM.

GSI34, a sulfonamide analogue and a small molecule inhibitor derived from GSIs (Figure 3), has been shown to prevent the induction of NICD by chemotherapeutic agents and inhibit *HES1* activation [9]. Another GSI, DAPT induced mitotic arrest in CRC cells in combination with taxanes [61]. DAPT was also used in another study that included GSIs such as L-685,458 and Dibenzazepine (DBZ) for their effect on CRC cell line growth or survival. Strikingly, this study was not able to elicit significant CRC cell growth inhibition using these GSIs. However, treatment with the compounds substantially reduced the abundance of the Val1744-NICD fragment (a Notch fragment that is highly detectable in a subset of CRC cells) within a few hours, with the effect from DBZ treatment being more pronounced and persistent. This result suggests that GSIs alone are not as potent in CRC but, in combination with chemotherapy, may be effective. In the same study, a combination of GSIs and platinum-based therapy, specifically cisplatin, was able to induce significant cell death in CRC [62]. LY3039478, an oral Notch signaling inhibitor in advanced or metastatic cancer, has been shown to be comparatively safe, and preliminary antitumor activity as a single agent has been recently demonstrated in CRC patients [63]. A recent study has demonstrated that the inactivation of DLL1- and DLL4-mediated Notch signaling resulted in a loss of intestinal goblet cells, but inducible deletion of Jagged1 has no overt phenotype [64]. Jagged1 knockdown reduced the migration and invasive capacity of the CRC cells in vitro and reduced tumor burden in a xenograft mouse model in vivo.

The unwanted side effects associated with GSI usage and the nonspecific nature of HES1 inhibitors are the major obstacles preventing these inhibitors from entering clinics. Thus, plant-derived natural compounds that may have low toxicity profiles are being explored as Notch1 inhibitor.

## 7. Natural Compounds Target Notch1 Activation

Dietary agents that inhibit Notch are considered ideal therapeutic agents and are being heavily investigated owing to their low toxicity, high therapeutic index, and better bioavailability potential. Small molecule inhibitors derived from compounds such as isoflavone genistein [65] found in soy products; sulforaphane derived from broccoli [66]; quercetin found in many fruits and vegetables [67]; curcumin used as a flavoring agent [67,68]; and resveratrol found in grapes [69], peanuts, and some berries, have been found to have some type of Notch-inhibiting activity that may be used to treat both solid tumors as well as cancer stem cells (CSCs). In one of our studies, we demonstrated that a small molecule inhibitor Verrucarin J (VJ) derived from natural sources successfully suppressed Notch1-mediated epithelial to mesenchymal transition in metastatic colon cancer [11]. Withaferin A (WA), another natural compound, has been shown to inhibit colon cancer cell growth by blocking signal transducer and activator of transcription 3 (STAT3) transcriptional activity [70]. We have also demonstrated the chemopreventive effect of WA on spontaneous and inflammation-associated colon carcinogenesis models. WA inhibited the expression of inflammatory (interleukin-6, tumor necrosis factor-alpha, and cyclooxygenase-2) and pro-survival (*pAKT*, Notch1, and nuclear factor kappa-light-chain-enhancer of activated B cells; *NF-κB*) markers in APCMin/+ and AOM/DSS models [10]. In another study conducted in our lab, through use of a natural compound, Psoralidin inhibited Notch1-mediated EMT activation in Aldehyde dehydrogenase positive (ALDH+) and ALDH− breast cancer tumors [71]. Although these inhibitors are considered comparatively safe to use, a detailed multimodal investigation of their efficacy and toxicity would be required before their approval as first-line therapies in colorectal cancer patients.

## 8. Targeting Downstream Notch1 Signaling Activators

Although the effects of the GSIs and natural molecules have shown some potential, the lack of target specificity warrants the use of more specific treatment strategies. Targeting pathways that are directly downstream of Notch are another viable treatment option. CRC cells that over-expressed *HES1* were more resistant to 5-Fluorouracil (5-FU) treatment in vitro [72]. Further, *HES1* regulates the invasion ability through the STAT3-MMP14 pathway in CRC cells, and high *HES1* expression is a predictor of poor prognosis of CRC [73]. HES1 expression in stage II CRC patients is correlated with a higher recurrence rate of the disease after chemotherapy [72]. Specific inhibitors of *HES1*, such as JI051 and its derivative JI130, were tested on pancreatic cancer cell lines and in xenograft experiments, showing suppression of cell growth and cell cycle arrest in vitro and a significant decrease in tumor growth in vivo [74]. Although this report is in pancreatic cancer, specific targeting of the *HES1*-repressive complex can also be applied to many other diseases, including CRC. Inhibitors of *HES1*, such as Crenigacestat (LY3039478), have provided evidence of clinical activity in heavily pretreated cancer patients [63].

Lenti-viral encoding Notch1 SiRNA significantly decreased Notch1 expression that resulted in the inhibition of cell growth and also caused cell cycle arrest at the G1 phase by inducing *P21* and P53 Up-Regulated Modulator of Apoptosis (*PUMA*) expression in CRC cells [75]. Silencing the Notch1 gene by SiRNA promoted docetaxel-induced cell growth inhibition, apoptosis, and cell cycle arrest in prostate cancer cells [76]. Zhang et al. [77] showed that the silencing of Notch1 enhanced the irradiation-induced cell proliferation inhibition and improved the radiosensitivity effect on CRC cells. The use of Notch1 SiRNA in combination with prevalent chemotherapeutic options has consistently been shown to be a better treatment option for different cancers [78].

As GSIs fail to discriminate between individual Notch receptors, monoclonal antibodies are used that specifically antagonize each receptor paralogue and thus easily differentiate the functions of Notch1 versus Notch2 in human patients and rodent models. Specific blocking of Notch1 activation with therapeutic antibodies such as mAb WC613 targeting the EGF-repeat region or OMP-52M51 targeting the LNR and HD region of Notch1 have shown significant efficacy for in vitro as well as in vivo cancer models [79]. Studies have demonstrated the complexity of Notch1 protein expression in human solid tumors and reiterated that Notch1 expression in tumorigenesis is highly context-dependent [80]. The use of mAbs that can be highly mutant-selective and that are also context-dependent inhibitors presents a unique and efficient strategy for Notch1 inhibition. However, treatments including the use of GSIs, and also mAbs, are not without their limitations, which we will discuss in the upcoming section.

## 9. Limitations of GSIs and mAbs in Notch1 Treatment

Although GSIs are considered valuable treatment options in combination with chemotherapeutic drugs, they are often not specific and block the processing of many other transmembrane proteins [81]. They must be given intermittently due to dose-limiting on-Notch toxicities [82]. The systemic use of the currently available γ-secretase inhibitors is associated with various adverse effects [83]. For example, the systemic use of LY411,575 in mice was associated with a significant loss of immature T cells [19] and an impairment of the development of lymphoid cells [84], as well as the damaged regenerative ability of colonic epithelial cells [19]. Other severe gastrointestinal tract toxicity, such as massive diarrhea as a result of a marked increase in goblet cell differentiation, has also been reported [85].

These limitations of GSIs make mAbs all the more promising; however, most of the mAb treatment regimens targeting the Notch1 extracellular domain or Notch1 ligands have shown limited efficacy in human clinical trials. For example, OMP-52M51, in a phase I dose-escalation trial (NCT01778439), showed limited antitumor efficacy [86], while a clinical trial for OMP-21M18/Demcizumab was stopped due to a lack of clinical responses [87].

Hence, recently, the focus has shifted to targeting different domains of Notch1 by developing antibodies designed to recognize the NRR region and thereby prevent the ADAM-mediated metalloprotease cleavage. Although receptor/ligand interactions were prevented by LBD-based antibodies in biochemical assays, these antibodies failed to inhibit the same interaction in cell-based assays. Meanwhile, antibodies that specifically target NRR were able to inhibit Notch signaling by downregulating the immediate Notch target gene Hes5 in both mouse and human stem cell systems [88]. Moreover, the small number of antibodies targeting the NRR region of Notch1 mostly bind to epitopes on one face of the NRR (LNR/HD region), which has emphasized the importance of this Notch-specific structural domain as the key to understanding how the activation of Notch1 is normally regulated [89]. The importance of this region in ligand-independent Notch1 activation, coupled with observations that all strong modulatory antibodies demonstrated binding with NRR, has generated enough interest in developing a potent and selective antibody and small molecule modulators of NRR.

## 10. Is NRR-Notch1 an Ideal Target for Therapy?

The first proteolytic cleavage during ligand-dependent Notch activation occurs at a site called S2, which lies within the NRR region of Notch. This cleavage prepares the Notch receptor for additional cleavages by the γ-secretase complex. Although ligand-induced activation of Notch signaling that involves a conformational change of the NRR is considered a major activating pathway, the activating mutations in the NRR and/or the PEST domain of Notch1 are seen in over 50% of cancer cases [43]. Mutations in the HD domain of the NRR are known to cause constitutive activation of Notch1 whilst having no effect on the chemical stability of Notch2 [43]. Natalie et al. (PMID: 26288744) investigated the effect of six mutations in NRR1 and NRR2. Five of the six mutations were in the HD domain of NRR and affected the ligand-dependent signaling of Notch, without affecting ligand-independent signaling. These studies indicated the need to develop specific antibodies for NRR to inhibit ligand-dependent or independent Notch signaling.

Wu et al. developed a co-crystal structure of NRR and demonstrated that stabilizing NRR quiescence can inhibit Notch1 signaling [79]. Receptor-directed antibodies are being developed that can antagonize Notch1, 2, and 3 by recognizing the NRR region of Notch, thereby preventing ADAM-mediated metalloprotease cleavage [89]. Dose-dependent inhibition of Notch1 signaling, without induction (-Jag1) or in the presence of a GSI (DAPT), was demonstrated by the antibodies that are specific for the NRR region of Notch1 [79]. The addition of purified NRR1 but not NRR2 antigen rescued signaling inhibited by anti-NRR1, confirming that inhibition reflected the specific binding of anti-NRR1 to NRR1. Although ∼45% sequence similarity exists between NRR1 and NRR2, the epitope residues are only 29% similar, elucidating the basis of anti-NRR1 specificity for Notch1 over Notch2. Specific monoclonal antibodies targeting Notch1 signaling in the breast and colon cancer cell lines have been developed; these mutant specific mAbs (604.107 and 604.164) impeded the growth of xenografts from breast and CRC cells and potentiated regression of the tumors when used in combination with Doxorubicin [90]. Antibodies targeting both normal and mutated NRR have been developed [91] and have been shown to be potent inhibitors of Notch1 signaling. More recently, secretory expression of NRR1 has been achieved in *Escherichia coli*, and a convenient model for preparing a functional polyclonal antibody has been established [92]. In a recent study in our lab, we developed a small molecule, ASR490, that binds with Notch1 NRR, as shown by molecular docking and CETSA studies. The binding of ASR490 stabilizes the NRR. The binding of ASR490 with NRR resulted in significant inhibition of activated Notch1 signaling and abrogated CRC cell growth and tumor growth in xenograft models (data not published). Targeting the NRR of the Notch receptor undoubtedly presents an interesting and specific immune-therapeutic option that can be utilized to inhibit ligand-dependent as well as independent Notch1 signaling.

These studies and an ever-evolving landscape of experimental data suggest that development of antibodies that selectively modulate the activities of NOTCH1 constitute a viable and convenient possibility. Even in a disease setting wherein most cell types express multiple Notch receptors, the capacity to activate and/or inhibit specific Notch receptors, either individually or in combination, would allow for the effective management of disease and also enable an understanding of their functional interrelationships. It is also highly possible that, in the near future, NRR-based antibody inhibitors will limit the toxicities associated with γ-secretase inhibitors, such as secretory diarrhea. Further development and characterization of antibody modulators of Notch activity are thus likely to have a broad experimental and therapeutic impact.

## 11. Conclusions and Future Directions

Notch1 signaling is necessary to maintain intestinal homeostasis. However, an aberrant activation (ligand-dependent or independent) disrupts the dynamic balance of Notch1-mediated regulatory pathways that eventually leads to the promotion and proliferation of CRC. Better characterization of these pathways may facilitate the development of Notch inhibitors that are mutant-selective and context-independent. This improved understanding will generate more effective GSIs, mAbs, and inhibitors specifically targeting the NICD complex. Furthermore, the development of small-molecule inhibitors or mAbs that specifically target a Notch isoform or stabilize the NRR of Notch1 will circumvent such issues as off-target toxicities associated with the chronic inhibition of wild-type Notch1. Another important aspect that needs attention is the extensive crosstalk of Notch with major oncogenic pathways such as *RAS*, *AKT*, and *NF-κB*. Better characterization of these pathways and the crosstalk between them that contributes to CRC pathogenesis is helping scientists to develop better therapeutic treatment regimens for CRC patients. Currently, there is growing evidence and optimism that the therapeutic targeting of Notch1 will become a mainstay of CRC treatment. It appears that the hurdles in successfully bringing Notch1 inhibition to the clinic will be overcome in the near future.
